# Cardiomyocyte oxidants production may signal to *T*. *cruzi* intracellular development

**DOI:** 10.1371/journal.pntd.0005852

**Published:** 2017-08-23

**Authors:** Patrícia Pereira Dias, Rhayanne Figueiredo Capila, Natália Fernanda do Couto, Damían Estrada, Fernanda Ramos Gadelha, Rafael Radi, Lucía Piacenza, Luciana O. Andrade

**Affiliations:** 1 Departamento de Morfologia, Universidade Federal de Minas Gerais, Minas Gerais, Brazil; 2 Departamento de Bioquímica, Center for Free Radical and Biomedical Research, Facultad de Medicina, Universidad de la República, Montevideo, Uruguay; 3 Departamento de Bioquímica e Biologia Tecidual, Instituto de Biologia, Universidade de Campinas, São Paulo, Brazil; Albert Einstein College of Medicine, UNITED STATES

## Abstract

Chagas disease, caused by the protozoan parasite *Trypanosoma cruzi*, presents a variable clinical course, varying from asymptomatic to serious debilitating pathologies with cardiac, digestive or cardio-digestive impairment. Previous studies using two clonal *T*. *cruzi* populations, Col1.7G2 (*T*. *cruzi* I) and JG (*T*. *cruzi* II) demonstrated that there was a differential tissue distribution of these parasites during infection in BALB/c mice, with predominance of JG in the heart. To date little is known about the mechanisms that determine this tissue selection. Upon infection, host cells respond producing several factors, such as reactive oxygen species (ROS), cytokines, among others. Herein and in agreement with previous data from the literature we show that JG presents a higher intracellular multiplication rate when compared to Col1.7G2. We also showed that upon infection cardiomyocytes in culture may increase the production of oxidative species and its levels are higher in cultures infected with JG, which expresses lower levels of antioxidant enzymes. Interestingly, inhibition of oxidative stress severely interferes with the intracellular multiplication rate of JG. Additionally, upon H_2_O_2_-treatment increase in intracellular Ca^2+^ and oxidants were observed only in JG epimastigotes. Data presented herein suggests that JG and Col1.7G2 may sense extracellular oxidants in a distinct manner, which would then interfere differently with their intracellular development in cardiomyocytes.

## Author summary

Chagas disease, caused by the protozoan parasite *Trypanosoma cruzi*, presents a variable clinical course, varying from asymptomatic to serious debilitating pathologies with cardiac, digestive or cardio-digestive impairment. It has been suggested that parasite differential tissue tropism is responsible for the development of the distinct clinical forms. Differences in parasite tissue tropism have been shown previously, using mixed infections in mice with two distinct *T*. *cruzi* populations, Col1.7G2 (*T*. *cruzi* I) and JG (*T*. *cruzi* II). In these infections hearts were preferentially colonized by JG. Increased JG adaptation to cardiac muscle was later confirmed in infection studies using isolated cardiomyocytes, where it was shown that selection was dependent on parasite intracellular development. However the mechanisms that determined this differential parasite intracellular growth was not described. Here we investigated whether host cell response upon *T*. *cruzi* infection was able to modulate parasite multiplication rate inside cells. We showed that, upon infection, cardiomyocytes increase the production of oxidative species, especially in cultures infected with JG and inhibition of oxidative stress severely interfered with the intracellular multiplication rate of JG. Data obtained suggests that JG and Col1.7G2 may sense extracellular oxidants in a distinct manner, which would enable JG to develop better inside cardiomyocytes.

## Introduction

Chagas disease, caused by the protozoan *Trypanosoma cruzi*, is an important health problem affecting about 6 to 7 million people worldwide [1]. Infection in man is defined by two distinct clinical phases. The acute phase, corresponding to the initial period of infection, is characterized by high parasitemia and tissue parasitism, followed by the chronic phase of the infection, which persists throughout the life of the host and is characterized by low tissue parasitism as well as parasitemia [2]. Chronic infection has a variable clinical course, ranging from asymptomatic cases (indeterminate form), to severe clinical conditions with heart (chagasic cardiomyopathy) and / or digestive tract (megacolon or megaesophagus) maladies. In patients with cardiac and / or digestive disorders, symptoms may appear between 10 and 30 years after initial infection and are due to the persistence of parasites in specific tissues, such as cardiac and / or smooth muscle, with the development of an intense inflammatory process, deleterious to the organ (reviewed by [3]).

Chagas disease clinical variability is well known to depend not only on genetic factors of the parasite, whose population structure is quite variable, but also on genetic factors of the host [4–6]. Previous studies conducted by our group showed that distinct parasite populations are found in different organs of infected patients [7], reinforcing data on the existence of a differential tissue tropism, probably related to the development of the diverse clinical forms [8–10]. Later, we studied this tissue tropism by performing mixed infections in BALB/c mice with two clonal populations of *T*. *cruzi*, Col1.7G2 (*T*. *cruzi* I) and JG (*T*. *cruzi* II), and detection of parasites directly from infected tissues. A predominance of Col1.7G2 was found in the rectum, diaphragm, esophagus and blood while JG was predominant in the cardiac muscle [11]. Later, we showed that this tissue tropism could be influenced by the genetic background of the host, where mice with the same MHC haplotype presented the same selection profile of *T*. *cruzi* in different tissues [12, 13]. *In vitro* studies using infection in cultures of cardiac explants or primary cardiomyocytes, with Col1.7G2 and JG, indicated that tissue selection occurs due to the direct interaction between parasite and host cell, without direct influence of the host immune system [13, 14]. In these studies, a more accelerated and efficient intracellular development of JG with respect to clone Col1.7G2 was observed in explants and cultures of cardiomyocytes isolated from BALB/c, suggesting that not only invasion, but also and mainly intracellular multiplication is important to tissue selection. Additionally, it was shown that this behavior profile was dependent on the cell type studied [14]. These findings reinforce that not only the parasite, but also the host cell response to infection is involved in the differential tissue tropism of *T*. *cruzi*. However, the mechanisms that define this selection are still poorly understood.

During cell infection, infective trypomastigotes adhere to the surface of the host cell, being internalized in parasitophorous vacuoles, formed by lysosomal membrane [15]. Trypomastigote later escape from the vacuole to the cytoplasm of the cell and turn into the amastigote replicative form, colonizing the host cell [16–18]. Thus, during cell infection parasite passes through different environments, which can directly or indirectly influence its behavior within the cell. Data from the literature show that infected cells are able to respond to infection by activating several genes, through the production of cytokines and reactive oxygen species (ROS), which could interfere with parasite intracellular behavior [19–24]. Here we investigate how stress responses mediated by oxidants in cardiomyocyte may influence infection by *T*. *cruzi* clonal populations, JG and Col1.7G2, interfering with their intracellular multiplication rates.

## Materials and methods

### Ethics statement

This study was carried out in strict accordance with the recommendations of the Guide for the Care and Use of Laboratory Animals of the Brazilian National Council of Animal Experimentation (http://www.cobea.org.br/) and Federal Law 11.794 (October 8, 2008). The institutional Committee for Animal Ethics of UFMG approved all the procedures used in this study. (CEUA/UFMG–Licenses 45/2009 and 261/2016)

### Parasites and cells

Two clonal populations of *Trypanosoma cruzi* were used, Col1.7G2 and JG, belonging to *T*. *cruzi* lineages I and II, respectively. JG strain was originally isolated in 1995 by Professor Eliane Lages-Silva (UFTM) from a chronic patient with megaesophagus. Col.1.7G2 is a clone from Colombian strain, which was originally isolated by Federici in 1969 from a chronic patient with cardiac disorders. Both *T*. *cruzi* populations were previously analyzed and characterized as monoclonal, through the analysis of the eight microsatellite loci according to previously described methodology [25].

Epimastigote forms of Col1.7G2 and JG were maintained in LIT (Liver Infusion Tryptose) medium containing 20 mg/mL of hemin and supplemented with 10% Fetal Bovine Serum and 1% Penicillin/Streptomycin, in T-25 cm^3^ bottles, in a 28°C incubator, and subcultured every two days [26].

Tissue culture trypomastigotes (TCTs) from Col1.7G2 and JG were obtained from the supernatant of infected LLC-MK2 monolayers and purified as described previously [27].

Primary cultures of cardiomyocytes were prepared from hearts of BALB/c mice neonates (0–2 days), according to protocol previously described [28]. 2x10^5^ purified cardiomyocytes were plated in each well of a 24 well pate, containing a 13 mm circular glass coverslips, and maintained in a 37°C CO_2_ incubator for 72 hours prior to infection. Alternatively, 5x10^4^ cells were plated in each well of a XF-24 cell culture microplate (Seahorse Bioscience) and maintained in a 37°C CO_2_ incubator for 120 hours prior to infection.

Cultures of human cardiomyocytes (Pluricardio) were prepared from the differentiation of induced pluripotent stem cells (iPSC) obtained from Pluricell Biotechnologies. Cells were thawed and plated after counting by trypan blue exclusion method at a confluence of 2x10^5^ cells per well in a 24-well plate. Cell differentiation was performed according to the company’s procedure, in 37°C CO_2_ incubator. All reagents required for maintenance and differentiation of cultures (extracellular matrix and culture media) were provided by the company. Five days after plating, after complete cell differentiation, cultures were used for the infection experiments.

Immortalized embryonic fibroblasts were originally isolated from C57BL/6 mouse embryos and spontaneously immortalized in culture [29]. These cells were maintained in culture by consecutive passages in 25 cm ^3^ flasks in DMEM (GIBCO) culture medium supplemented with 10% FBS and 1% antibiotic (penicillin/streptomycin). For the infection assays, 8x10^4^ cells were plated in each well of a 24-well plate containing a 13 mm circular glass coverslips and maintained at 37°C in a CO_2_ incubator.

### Treatments

For some experiments, parasites (epimastigote stage) were previously treated with H_2_O_2_. H_2_O_2_ solutions were prepared daily assuming an extinction coefficient of 81M^-1^ cm^-1^ at 230 nm. For this, epimastigotes were subjected to treatment with different concentrations of H_2_O_2_ (5 to 100 μM) for 6 days when the number of parasites was counted. Since 30 μM of H_2_O_2_ was the highest concentration in which JG and Col1.7G2 growth was still observed we used this concentration to perform the experiments. Treatment was performed by incubating 10^7^ epimastigotes in 1mL PBS, pH 7.3 in the absence (control) or presence of 30 μM H_2_O_2_ at 28°C, for 30 minutes. Parasites were then washed and re-suspended in specific medium according to the experiment to be performed.

For some experiments, cardiomyocyte cultures were treated with catalase (Catalase-PEG C4963—SIGMA) at a final concentration of 40U/mL. Treatment was initiated 2 hours prior to infection and maintained during and after parasite exposure.

### Cell infection assay

Cardiomyocyte cultures pre-treated or not with catalase, as well as fibroblast cultures were exposed to purified JG or Col1.7G2 TCTs re-suspended in high-glucose DMEM at a multiplicity of infection (MOI) of 50. The infection was performed for 40 minutes at 37°C. After infection, monolayers were washed at least four times with PBS and either fixed with 4% (w/v) paraformaldehyde in PBS (0h–to determine the rate of parasite invasion) or re-incubated for 4, 8, 12, 24, 48 or 72 hours prior to fixation and processing for immunofluorescence assays (to determine parasitophorous vacuole escape– 4, 8 and 12 h or parasite intracellular multiplication rates– 24, 48 and 72 h). Alternatively, after parasite exposure (40 minutes at 37°C), cultures were washed and re-incubated for 48 and 72 hours before ROS measurements.

### Immunofluorescence

After treatment, infection and fixation, coverslips containing attached cells were washed with PBS, incubated for 20 minutes with PBS containing 2% (w/v) bovine serum albumin (BSA) (Sigma-Aldrich) and processed for an inside/outside immunofluorescence invasion assay as described previously [27]. Briefly, cells were fixed and extracellular parasites were immune-stained using a 1:500 dilution of rabbit anti-*T*. *cruzi* polyclonal antibodies in PBS containing 2% (w/v) BSA (PBS/BSA) followed by labeling with Alexa Fluor-546 conjugated anti-rabbit IgG antibody (Invitrogen).

For evaluation of parasitophorous vacuole escape, after extracellular parasite staining, cells were permeabilized using a solution containing 2% (w/v) BSA and 0.5% (v/v) saponin (Sigma-Aldrich) in PBS (PBS/BSA/saponin) for 20 minutes. Host cell lysosomes were then immunostained using a 1:50 dilution of rat anti-mouse LAMP-1 hybridoma supernatant (1D4B; Developmental Studies Hybridoma Bank, USA) in PBS/BSA/saponin for 45 minutes followed by labeling with Alexa Fluor-488 conjugated anti-rat IgG antibody (Invitrogen), as described previously [30]. Subsequently, DNA from both host cells and parasites were stained for 1 min with 10 μM of DAPI (Sigma-Aldrich), mounted on glass slide and examined on an Olympus BX51, Zeiss, Apotome or Nikon Eclipse Ti.

### Evaluation of oxidant production by infected cells

A general measure of oxidant formation were performed using CM-H_2_DCFDA (5- (and-6) -chloromethyl-2', 7'-dichlorodihydrofluorescein diacetate, acetyl ester—Molecular Probes) probe, which fluoresces upon oxidation. For this, cell cultures, 48 and 72 hours post infection with JG or Col1.7G2, were washed once with PBS and exposed to CM-H_2_DCFDA at a final concentration of 10 μM in PBS. Immediately after the addition of CM-H_2_DCFDA, the plate was read on a Varioskan Flash (Thermo Scientific) at 37°C for monitoring the probe’s oxidization rate, with excitation and emission wavelengths of 485 and 520 nm, respectively. The data were analyzed using the program SkanIt Software 2.4.5. The probe oxidation curves were used to calculate the slope and are expressed as Relative Fluorescence Units (RFU)/min.

### Cell respiration assay

Neonatal cardiomyocyte cultures plated in an XF-24 well culture microplate (Seahorse Bioscience) were infected with TCTs from JG or Col1.7G2, as described above for 48 hours. One hour before the experiment, media was replaced with unbuffered Dulbecco’s Modified Eagle Medium (DMEM, pH 7.4) supplemented with 4 mM L-glutamine, 5 mM glucose and 10 mM Pyruvate (Gibco). Olygomycin (5 μM, an inhibitor of ATP synthase (complex V)), Carbonyl Cyanide-p-trifluoromethoxyphenylhydrazone (FCCP 5 μM, uncoupling agent) and a mix of antimycin A (AA, complex III inhibitor) and rotenone (Complex I inhibitor) at a final concentration of 5 and 1 μM, respectively were injected sequentially through ports in the seahorse flux pack cartridges. Oxygen consumption rates (OCR) were analyzed for control and infected cardiomyocytes [31]. At least 5 replicates of each condition per plate and three independent replicates were analyzed. The non-mitochondrial oxygen consumption obtained after AA/Rotenone addition were subtracted to all OCR values. The mitochondrial respiratory control index (RCI) was calculated as the OCR value with FCCP divided by the OCR value with oligomycin (FCCP/Oligomycin).

### Quantification of antioxidant enzymes from parasites

For the identification and quantification of anti-oxidant enzymes produced by the different *T*. *cruzi* populations, 1x10^7^ epimastigote forms previously incubated or not with 30 μM H_2_O_2_ (30 min) were fixed with 3.7% (v/v) formaldehyde in PBS, centrifuged at 12,000 g at room temperature (RT), resuspended in a solution containing 0.1% (v/v) Triton in PBS and incubated for 30 minutes at RT for permeabilization. After permeabilization, samples were centrifuged and incubated overnight at 4°C with a 1/100 dilution of each of the rabbit polyclonal antibodies raised towards the different antioxidant enzymes analyzed (Ascorbate peroxidase, APX; Mitochondrial Peroxiredoxin, MPX; Trypanothione reductase, TR; Trypanothione synthetase, TS; mitochondrial iron superoxide dismutase A, FeSOD-A and cytosolic iron superoxide dismutase B, FeSOD-B), in PBS containing 0.1% (w/v) BSA and 0.5% (v/v) Tween (PBS/BSA/Tween). After this, samples were centrifuged, washed in PBS/BSA/Tween and incubated for 90 minutes with Alexa-Fluor 488-labeled anti-rabbit IgG secondary antibody (anti-APX, MPX, TR and TS) or anti-mouse IgG (anti-SODA and SODB) diluted 1:100 in PBS/BSA/Tween. After incubation with the secondary antibody, the samples were centrifuged, washed with PBS/BSA/Tween, re-suspended in the same solution and read on BD FACSCan or BD FACSCalibur flow cytometer. Acquired data were analyzed using the BD CellQuest Pro 6.0 or FlowJo program.

### Determination of intracellular calcium concentrations, and superoxide production in epimastigotes

For intracellular calcium measurements, 5x10^7^ parasites/mL were loaded with 5 μM fura-2AM at 28–30°C in fura buffer (116 mM NaC1, 5.4 mM KC1, 0.8 mM MgSO_4_, 5.5 mM glucose, 1 mM CaC1_2_, and 50 mM Hepes, pH 7.0). After 1h, cells were washed and re-suspended in PBS and exposed or not to H_2_O_2_-treatment as described earlier. Afterwards cells were washed once in PBS, re-suspended in fura buffer and fluorescence determined in 10^7^ cells/mL in fura buffer in a Hitachi F2500 fluorescence spectrophotometer with continuous stirring (excitation at 340 and 380 nm and emission at 510 nm) [32, 33].

For determination of epimastigote oxidant production in control and/or after H_2_O_2_ treatment, parasites (3 x10^8^/mL) were loaded in Krebs-Henseleit buffer (KH buffer, 15 mM NaCO_3_, 5 mM KCl, 120 mM NaCl, 0.7 mM Na_2_HPO_4_, 1.5 mM NaH_2_PO_4_) at 28°C with 5 μM MitoSOX (3,8-phenanthridinediamine,5-(6-triphenylphosphoniumhexyl)-5,6-dihydro-6- phenyl, Molecular Probes). After 10 min of incubation with the probe, the cells were washed and re-suspended in KH buffer. Cells were then incubated with H_2_O_2_ for 30 min and washed, as described. The detection of oxidized MitoSOX (oxMitoSOX) in 5x10^7^ cells/mL was performed in this buffer in the presence of 40 μM digitonin and 5 mM succinate to determine the production of these species by the mitochondrial respiratory chain. The fluorescence was detected using a Cytation 5 microplate reader with excitation and emission wavelengths of 510 and 580 nm, respectively [34].

## Results

### JG develops better in BALB/C neonatal primary cardiomyocyte cultures

**Fig 1:**
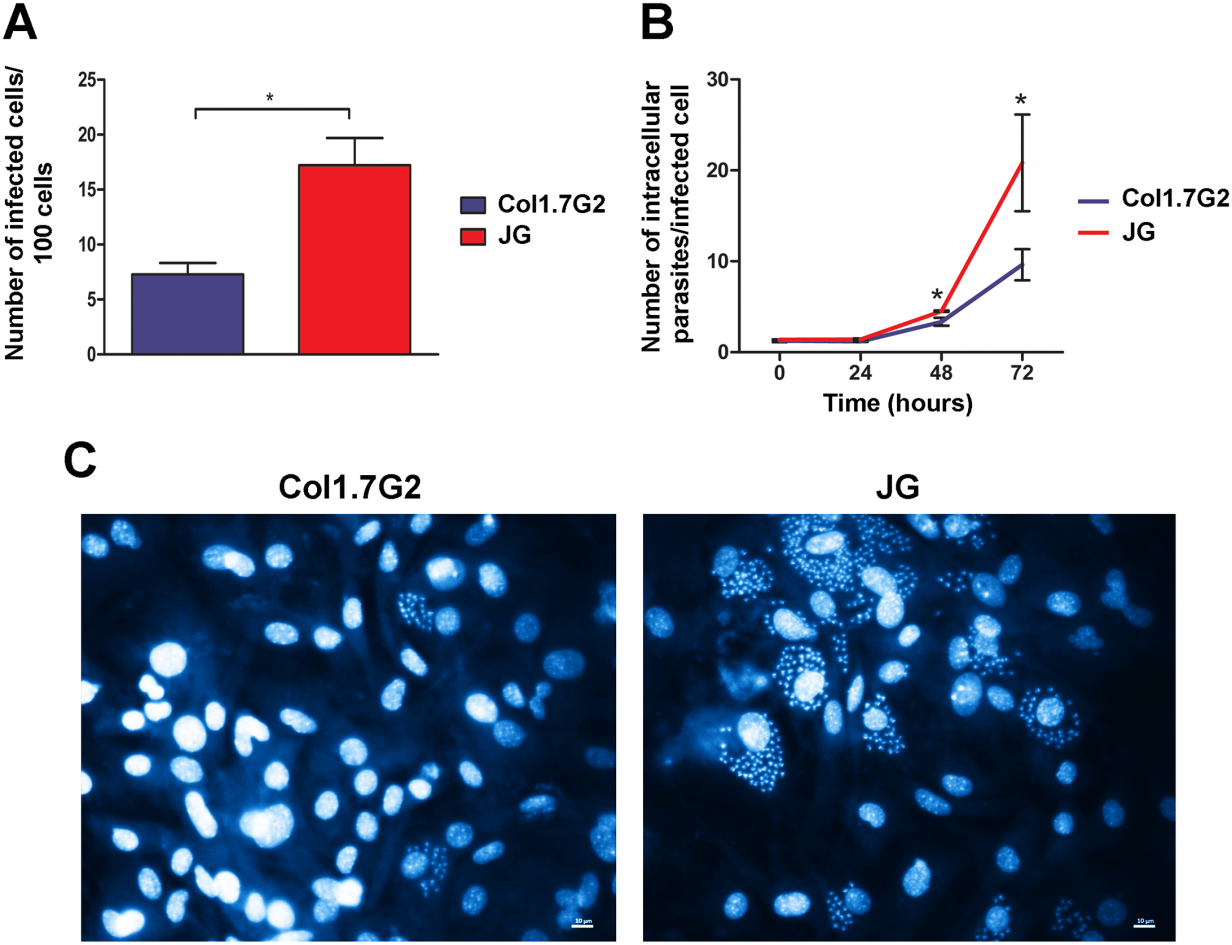
(A-B) Quantification of invasion and intracellular multiplication rates of Col1.7G2 and JG in primary cultures of cardiomyocytes. Primary cultures of cardiomyocytes from neonatal mice were exposed to Col1.7G2 or JG and the number of infected cells per 100 cells (A), as well as the number of intracellular parasites per infected cell (B), were evaluated in order to determine the invasion and intracellular multiplication rates, respectively. The data represent the mean of triplicates ± the standard error of the mean (SEM). Asterisks indicate statistically significant differences (* p≤0.05—Student's t-test). (C) Representative images of cell infection in primary cultures of cardiomyocytes from BALB/c neonatal mice, 72 hours after exposure to JG or Col1.7G2 (40X). The nuclear marker, DAPI, was used to identify the genetic material of both the parasite and the host cell. The scale bar corresponds to 10 μm.

In order to confirm previous results obtained from primary embryonic cardiomyocyte cultures infected with JG or Col1.7G2 [14], cultures obtained from neonatal BALB/c mice were submitted to infection with the same *T*. *cruzi* populations and invasion and intracellular multiplication rates were analyzed. A different behavior regarding cell invasion was observed. JG infection rate was in this case higher than Col1.7G2. The number of infected cells was about 4.5 times higher for cultures exposed to JG, when compared to cultures infected with Col1.7G2 (Fig 1A). Intracellular multiplication rates, on the other hand, were in accordance with previous results from Andrade and colleagues (2010) [14]. Seventy-two hours post infection we observed that the number of intracellular parasites in JG infected cultures were higher than the number of intracellular parasites in cultures infected with Col1.7G2 (Fig 1B). 72 hours after exposure to the parasites, cultures infected with JG showed an approximately 2.5-fold increase in the number of intracellular parasites relative to those cultures infected with Col1.7G2 (Fig 1B). Fig 1C shows representative images of the number of intracellular parasites in cardiomyocyte 72 hours post invasion with each of the parasite populations. These results indicate that independently of the invasion rate, JG shows a better intracellular development in these cells when compared to Col1.7G2 (as noted by the slope of the curve).

### JG and Col1.7G2 show the same parasitophorous vacuole escape kinetics

As it is known, when *T*. *cruzi* trypomastigotes invade the host cell it first resides in a parasitophorous vacuole, formed by lysosomal membrane, and later escapes from this vacuole falling into the host cell cytosol, where it transforms into the amastigote replicative form. Therefore the kinetics of parasitophorous vacuole escape could alter the transformation of internalized trypomastigotes into amastigote forms and consequently parasite intracellular development. In order to determine the kinetics of parasitophorous vacuole escape for JG and Col1.7G2 in primary neonatal cardiomyocyte cultures, infected cultures were washed and fixed at 0, 4, 8 or 12 hours after exposure to the parasites, as described. Parasites labeled with DAPI and lacking anti-*T*. *cruzi* antibody labeling were considered as intracellular parasites. To evaluate the proportion of intracellular parasites associated with the parasitophorous vacuole, the cells were also labeled with anti-LAMP-1 antibody, a protein present on the lysosomal membrane. Thus, intracellular parasites that co-localized with this marker were counted as inside the vacuole, the other intracellular parasites were considered free in the cytoplasm.

**Fig 2:**
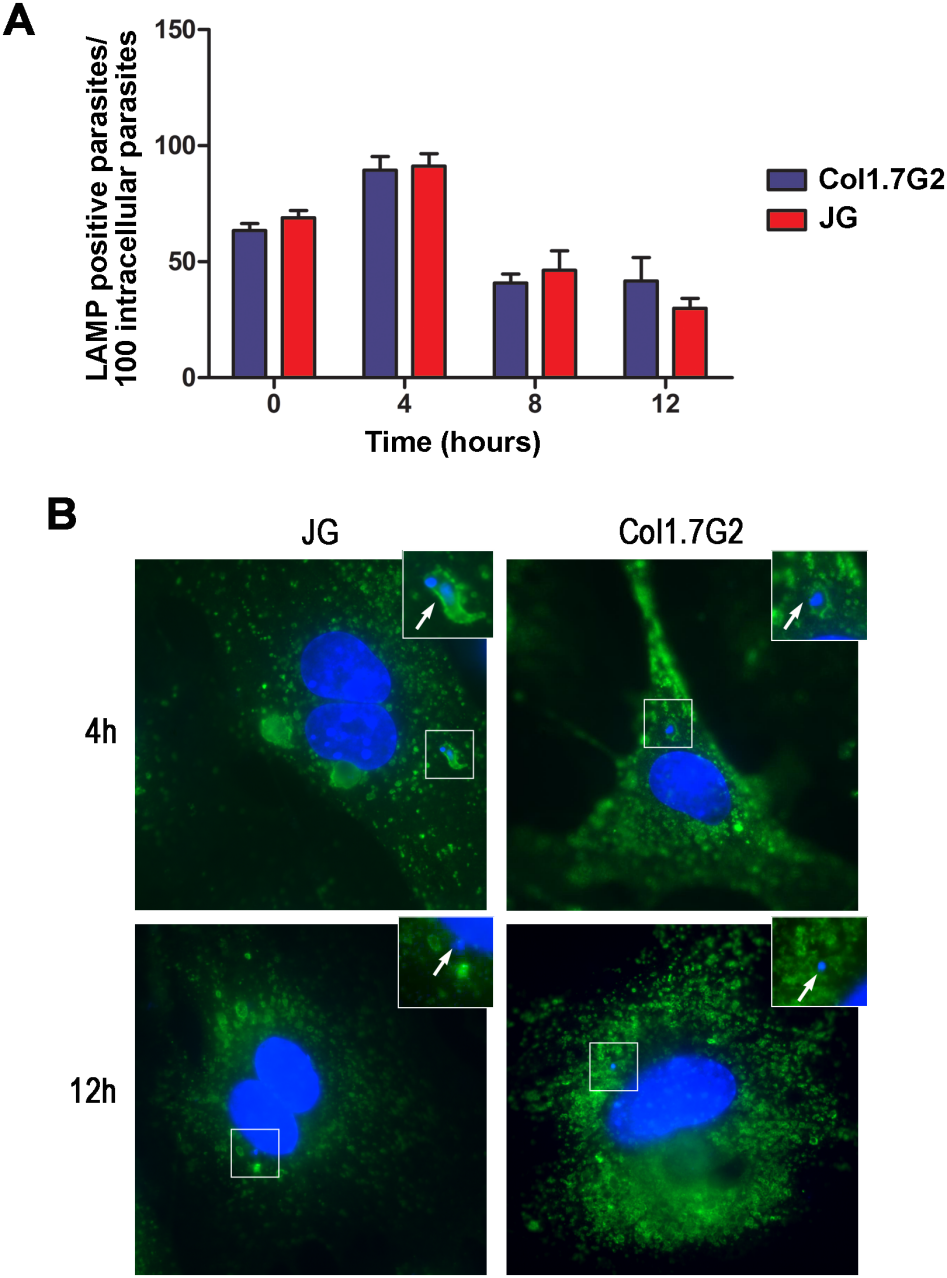
Parasitophorous vacuole escape rate for *T*. *cruzi* clonal populations, Col1.7G2 and JG, upon infection in primary mouse cardiomyocytes. (A) The graph shows the proportion of intracellular parasites associated with LAMP-1, a lysosomal marker, over the time of infection. The data represent the mean of triplicates ± the standard error of the mean (SEM). The data shown are representative of three individual experiments. (Student's t-test). (B) Representative images of intracellular parasites in primary cultures of cardiomyocytes from BALB/c neonatal mice, inside or outside the parasitophorous vacuole, 4 and 12 hours (respectively) after exposure to JG or Col1.7G2 (100X). The nuclear marker, DAPI, was used to identify the genetic material of both the parasite and the host cell and anti-LAMP-1, a lysosomal marker, was used to identify parasites inside vacuole. The scale bar corresponds to 10 μm.

The number of parasites associated with the lysosomal marker, LAMP-1, shortly after (0h), 4, 8 and 12 hours after cell exposure to parasites is shown in Fig 2. No significant difference was observed in the kinetics of vacuole escape between JG and Col1.7G2. Soon after the invasion, for both *T*. *cruzi* populations, around 50% of the internalized parasites are associated with LAMP (Fig 2A). Four hours after parasite removal, the number of parasites associated with LAMP reaches 100%. This is due to the fact that the abundance of lysosomal markers associated to the vacuole increases in the first moments after the invasion, facilitating its visualization. Later, 8 to 12 hours post invasion, the number of parasites associated with LAMP starts to drop for both JG and Col1.7G2 infections, indicating that the parasites are escaping from the vacuole into the cytosol (Fig 2A). Representative images of parasites inside (4 hours) or outside the vacuole (12 hours), for both JG and Col1.7G2, are shown in Fig 2B.

### JG-infected primary cardiomyocyte cultures produce more reactive oxygen species

In order to identify other possible factors that could account for the differential growth rate of JG and Col1.7G2 in neonatal cardiomyocytes, we evaluated the levels of production of oxidants in these cells upon infection with the two *T*. *cruzi* clonal populations. It is known that cardiomyocyte infection by the parasite can induce the production of ROS, which can modulate the intracellular development of the parasite [35, 36]. Analysis of the oxidant levels produced upon infection with JG and Col1.7G2 in cardiomyocyte cultures was done using the CM-H_2_DCFDA probe added to infected cultures, as described in material and methods. When oxidized, CM-H_2_DCFDA fluoresces and the amount of fluorescence produced is an indirect measure of the cellular production of ROS. CM-H_2_DCFDA fluorescence was measured 48 or 72 hours post infection, the period corresponding to the intracellular multiplication phase of the parasite.

**Fig 3:**
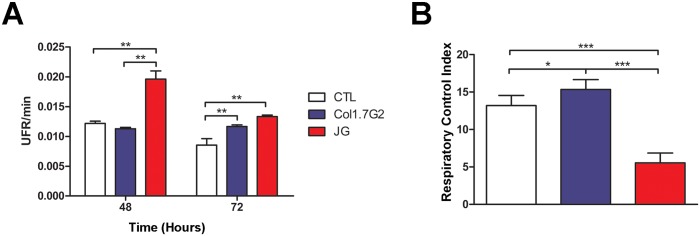
ROS production and mitochondrial function in JG and Col1.7G2 infected cardiomyocytes. (A) Relative fluorescence levels of oxidized CM-H_2_DCFDA present in primary BALB/c neonatal cardiomyocyte cultures, 48 and 72 hours after infection with Col1.7G2 or JG. Cultures of uninfected primary cardiomyocytes maintained for the same times were used as controls. The data represent the mean of duplicates ± the standard error of the mean (SEM). (B) Respiratory Control Index—RCI in primary cultures of BALB/c neonatal cardiomyocytes, infected with Col1.7G2 or JG, 48 hours after infection. Asterisks indicate statistically significant differences (* p<0.05, ** p≤0.01, *** p <0.001—Student's t-test).

Forty-eight hours post infection no significant difference in the amount of oxidized probe was observed for those cultures infected with Col1.7G2, relative to the control (uninfected cultures) (Fig 3A). On the other hand, at the same time the levels of CM-H_2_DCFDA oxidation were about 1.6 fold higher for JG infected cultures when compared to the control or cultures infected with Col1.7G2, indicating increased oxidant production after infection with JG (Fig 3A). At 72 hours, both JG and Col1.7G2 infected cultures showed significantly higher levels of CM-H_2_DCFDA oxidization when compared to control non-infected cultures. (Fig 3A). However, the levels of oxidized CM-H_2_DCFDA in JG-infected cultures were still significantly higher than that observed for cultures infected with Col1.7G2 (Fig 3A).

It had been shown that oxidant production by *T*. *cruzi* infected cardiomyocytes could come from mitochondrial dysfunction [23]. Thus, we evaluated the mitochondrial function in cultures of primary mouse cardiomyocytes infected or not with trypomastigote forms of Col1.7G2 or JG. The respiratory control index (RCI) allows the evaluation of the mitochondrial capacity of substrate oxidation with low proton loss. Thus, the higher the RCI the lower is mitochondrial dysfunction and oxidant production. RCI measurements 48 hours post infection revealed greater mitochondrial impairment in cultures infected with JG. While cardiomyocytes infected with Col1.7G2 showed a small increase in the RCI when compared to control non-infected cultures, cardiomyocyte cultures infected with JG showed a significantly lower RCI when compared to control or Col1.7G2 infected cultures (Fig 3B). These results are in agreement with the higher rates of CM-H_2_DCFDA probe oxidation, indicating greater mitochondrial dysfunction and consequently higher production of oxidizing species cardiomyocyte cultures infected with JG.

### JG epimastigote forms expresses lower amounts of antioxidant enzymes

**Fig 4:**
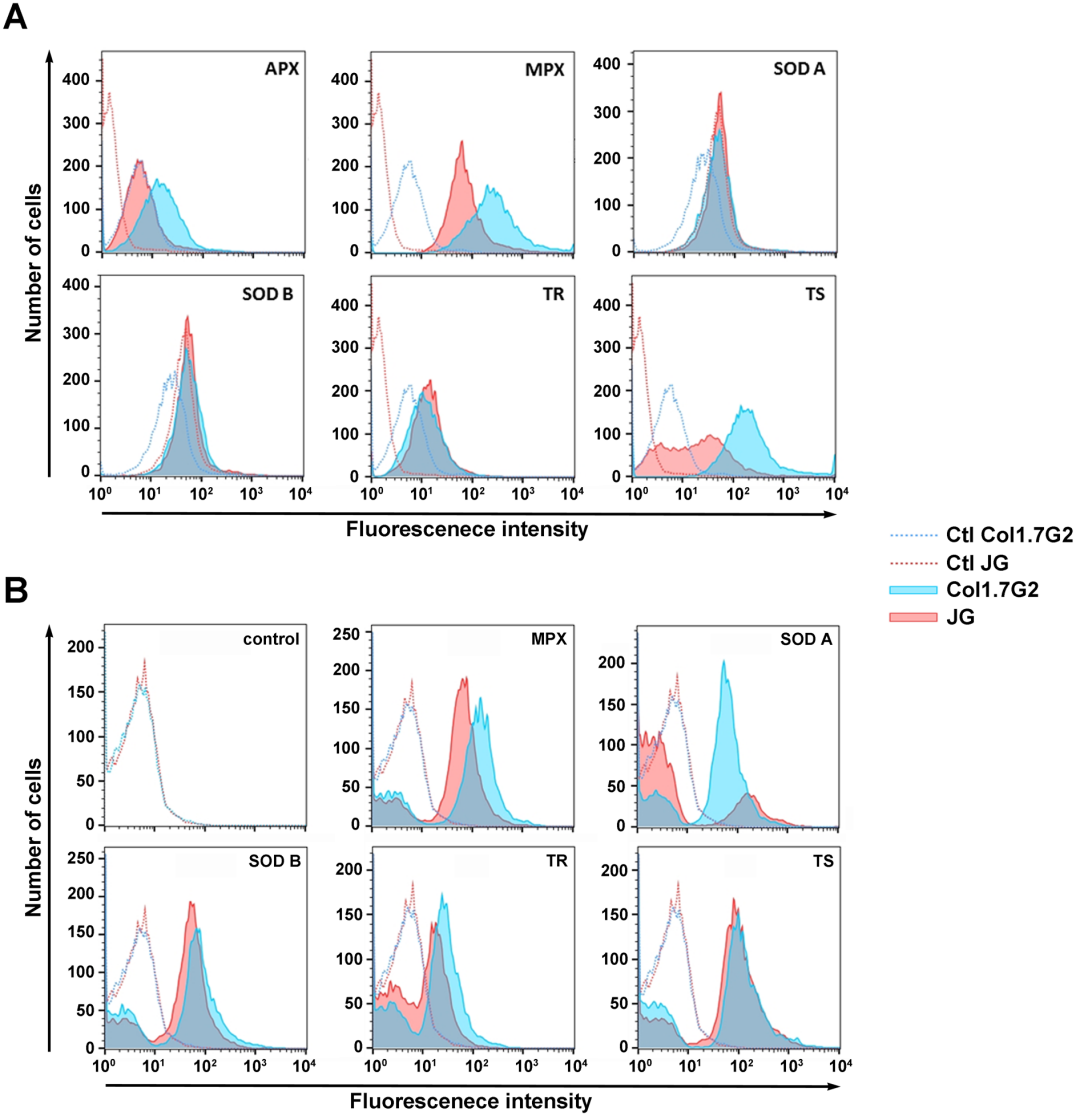
Mean fluorescence intensity obtained from labeling of epimastigote forms of the two *T*. *cruzi* clonal populations. Col1.7G2 (blue) and JG (red) with polyclonal antibodies against different *T*. *cruzi* anti—oxidant enzymes: Ascorbate peroxidase, APX; Mitochondrial Peroxiredoxin, MPX; Superoxide Dismutase A, SOD A; Superoxide Dismutase B, SOD B; Trypanothione Redutase, TR and Trypanothione Synthase, TS before (A) and after (B) treatment with 30 μM H_2_O_2_. Dotted lines represent control non-labeled parasites.

To assess the ability of JG and Col1.7G2 to cope with ROS produced upon infection in cardiomyocytes, the basal levels of different parasite anti-oxidant enzymes were assayed. Polyclonal antibodies directed to each of the anti-oxidant enzymes were used to label the parasites and the amount of labeling was read in a flow cytometer. Fig 4A shows the histograms of fluorescence intensities obtained in the epimastigote stage, for each *T*. *cruzi* population, for the different enzymes. The higher the expression of the enzymes the higher the number of cells presenting high levels of fluorescence. APX, MPX and TS anti-oxidant enzymes were found in higher amounts in the epimastigote forms of Col1.7G2 when compared to the same forms of JG in control conditions (Fig 4A).

To determine if the profile of the antioxidant enzyme production by JG and Col1.7G2 would be the same after exposure of the parasites to oxidative stress, epimastigote forms from JG and Col1.7G2 were treated with H_2_O_2_ prior to the evaluation of enzyme expression. Even after exposure to the oxidant, a higher content of antioxidant enzymes was found for epimastigote forms of clone Col1.7G2. In this condition, higher expression of MPX, Fe-SODA and TR were found in Col1.7G2 epimastigotes when compared to JG (Fig 4B). Therefore, higher amounts of anti-oxidant enzyme was only observed for Col1.7G2, never for JG, either before or after exposure to an oxidative environment, indicating that JG could be more susceptible to oxidative stress.

### Inhibition of host cell oxidative stress by treatment with catalase affects JG intracellular multiplication

**Fig 5:**
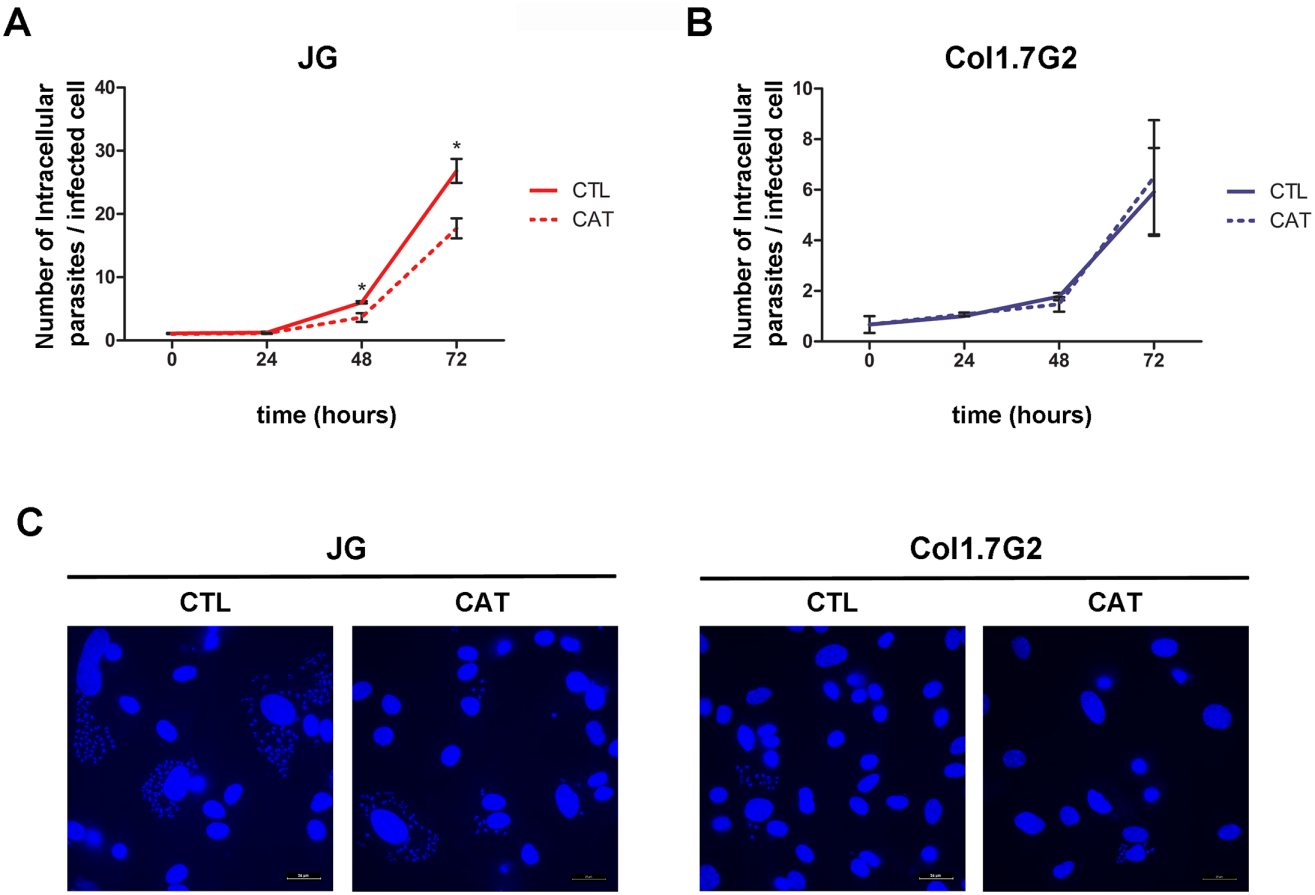
Quantification of intracellular multiplication rates of JG and Col1.7G2 in mouse cardiomyocyte cultures treated or not with catalase. (A and B) Primary BALB/c neonatal cardiomyocyte cultures were treated (dashed line) or not (continuous line) with catalase, exposed to JG (A) or Col1.7G2 (B) and the number of intracellular parasites per infected cell along 72 hours of infection was evaluated. The data represent the mean of triplicates ± the standard error of the mean (SEM). Asterisks indicate statistically significant differences (* p≤0.01—Student t test). (C) Representative images of cell infection in primary BALB/c neonatal cardiomyocytes cultures, treated with catalase, 72 hours after exposure to JG or Col1.7G2. The scale bar corresponds to 10 μm.

The results obtained above show that JG induces more ROS in infections of BALB/c neonatal cardiomyocyte cultures and has less anti-oxidant enzymes contents when compared to Col1.7G2. Nonetheless, intracellular multiplication of JG in these cells is faster when compared to Col1.7G2. These data suggest that, ROS production and oxidative stress generated during infection in cardiomyocytes may trigger JG intracellular development. To test this hypothesis we decided to investigate whether decreasing reactive oxygen species, such as hydrogen peroxide (H_2_O_2_) by treatment of cardiomyocyte cultures with catalase during *T*. *cruzi* infection, could interfere with the intracellular development of JG and or Col1.7G2 in these cells. For this, cultures of BALB/c neonatal cardiomyocytes were incubated or not with catalase, as described in the methodology. After treatment with catalase, a statistically significant decrease in the intracellular growth rate of JG was observed. JG infected cells had lower number of intracellular parasites along the course of infection when compared to non-treated cells (Fig 5A and 5C). Seventy-two hours post infection, the number of JG intracellular parasites for cultures treated with catalase was around 1.5 times lower than that obtained for control non-treated cultures, indicating a poorer intracellular development in a less oxidative environment (Fig 5A). On the other hand, treatment of cultures with catalase did not interfere with Col1.7G2 intracellular growth (Fig 5B and 5C), since the number of intracellular parasites along the course of infection was very similar in both treated and catalase treated conditions. These results suggest that the oxidative stress generated by the infection plays an important role in stimulating the intracellular development of the JG strain.

### The behavior of JG and Col1.7G2 can be reproduced in cultures of human cardiomyocytes

**Fig 6:**
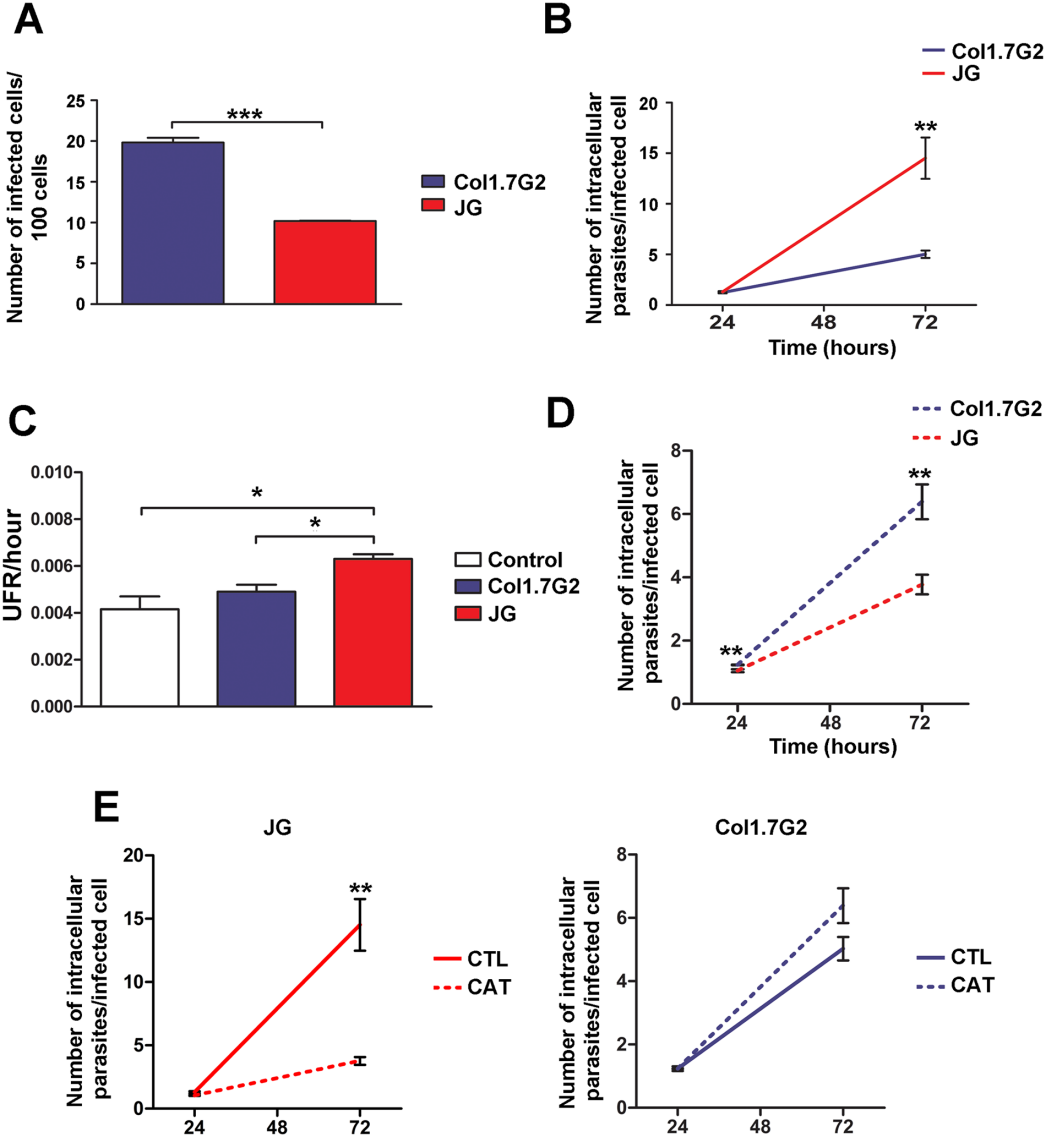
(A-B) Quantification of invasion and intracellular multiplication rates of Col1.7G2 and JG in human cardiomyocyte cultures. Cultures of human cardiomyocytes derived from iPSCs were exposed to Col1.7G2 or JG and the number of infected cells per 100 cells (A), as well as the number of intracellular parasites per infected cell (B), were evaluated for the determination of invasion and intracellular multiplication rates, respectively. The data represent the mean of triplicates ± the standard error of the mean (SEM). (C) Relative fluorescence of oxidized CM-H_2_DCFDA present in human cardiomyocyte cultures, 48 hours after infection with Col1.7G2 or JG. Cultures of uninfected human cardiomyocytes maintained for the same time were used as controls. The data represent the mean of duplicates ± the standard error of the mean (SEM). (D) Quantification of intracellular multiplication rates of Col1.7G2 and JG in cultures of human cardiomyocytes treated with catalase. Human cardiomyocyte cultures from iPSCs were treated with catalase and then exposed to Col1.7G2 or JG and the number of intracellular parasites per infected cell was evaluated. The data represent the mean of triplicates ± the standard error of the mean (SEM). (E) Combination of data from graphs shown in B and D, showing JG or Col1.7G2 intracellular growth from independent experiments performed in catalase treated (dashed lines) and non-treated (continuous lines) human cardiomyocyte cultures. Asterisks indicate statistically significant differences (*** p≤0.001, ** p≤0.01 and * p≤0.05—Student's t-test).

The data obtained for primary cultures of cardiomyocytes from BALB/c mice suggest that the oxidative stress generated during infection benefits the growth of JG in these cultures. In order to verify if this data could be reproduced in cardiomyocytes from a different source, we performed cultures of human cardiomyocytes obtained from induced pluripotent human stem cells. First, the cultures of human cardiomyocytes were submitted to the same infection methodology with Col1.7G2 and JG and the rates of invasion and intracellular multiplication were evaluated. In these cultures, the rate of invasion observed for the two *T*. *cruzi* clonal populations, JG and Col1.7G2, was similar to the results previously obtained by Andrade *et al*. (2010), where Col1.7G2 had a higher number of infected cells when compared to cultures infected with JG (Fig 6A). With respect to parasite growth, 72 hours post-infection, JG-infected cultures showed higher intracellular proliferation rates (2.14 times) when compared to cultures infected with Col1.7G2 (Fig 6B), reproducing the results obtained by Andrade *et al*. (2010) [14] and data obtained here for BALB/c neonatal cardiomyocyte cultures (Fig 1B). JG growth in human cardiomyocyte cultures was about 2.14 times greater than Col1.7G2 (Fig 6B).

### Oxidative stress generated during infection in human cardiomyocyte cultures also contribute to JG intracellular development in these cells

Since the profile of JG and Col1.7G2 intracellular development in human cardiomyocytes reproduced the data obtained from infections in neonatal BALB/c cardiomyocyte cultures, we decided to investigate the induction of oxidants upon infection of these cells. Evaluation of oxidant production was also performed by incubation of cells with the probe CM-H_2_DCFDA, 48 hours post infection. Again, no significant difference was observed in the amount of oxidized probe for those cultures infected with Col1.7G2, relative to the control (uninfected cultures) (Fig 6C). On the other hand, at the same time a significantly higher amount of probe oxidation, about 1.52 fold higher, was observed for those cultures infected with JG, relative to the control or about 1.29 fold higher when compared to cultures infected with Col1.7G2, also indicating higher production of ROS upon infection of these cells with JG (Fig 6C). Additionally, we also investigated whether inhibition of oxidative stress would affect JG or Col1.7G2 infection in these cultures. While JG multiplied better than Col1.7G2 in non-treated cultures (Fig 6B), its growth was lower than Col1.7G2 in catalase treated cultures (Fig 6D). We also compared JG and Col1.7G2 intracellular growth obtained from experiments performed in human cardiomyocyte non-treated cultures (Fig 6B) with the new data obtained from the experiments performed with human cardiomyocytes treated with catalase (Fig 6D), which are shown in Fig 6E. As observed, a decrease in JG intracellular growth, but not in Col1.7G2 is found upon catalase treatment. These results imply that JG is also more responsive than Col1.7G2 to the repressive effects of catalase when infecting human cardiomyocyte cultures and reinforce the idea that the oxidative stress generated by the infection plays an important role in the intracellular development of at least some *T*. *cruzi* strain.

### Infection in immortalized mouse fibroblasts does not generate oxidative stress and the intracellular growth profiles of JG and Col1.7G2 are similar

**Fig 7:**
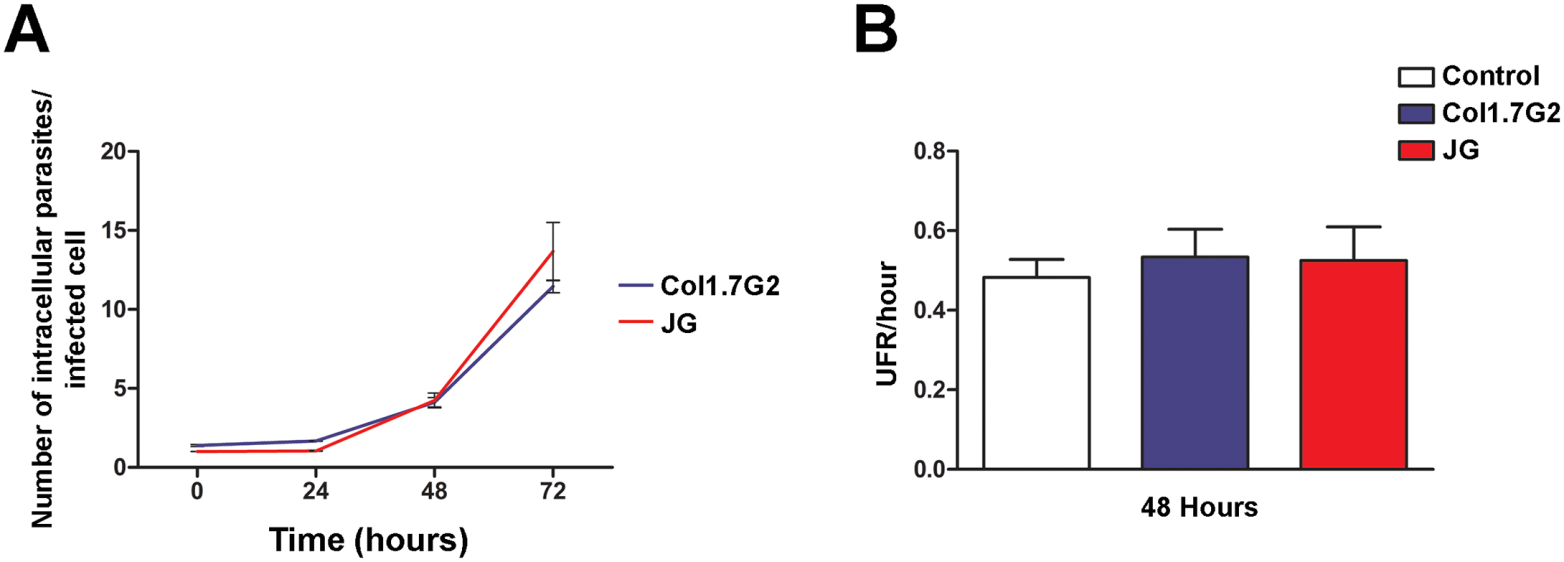
(A) Quantification of intracellular multiplication rates of Col1.7G2 and JG in mouse embryonic fibroblasts (MEFs) cultures. Cultures of MEFs were exposed to Col1.7G2 or JG and the number of intracellular parasites per infected cell was evaluated. The data represent the mean of triplicates ± the standard error of the mean (SEM). (B) Relative fluorescence of oxidized CM-H_2_DCFDA present in MEFs cultures, 48 hours after infection with Col1.7G2 or JG. Cultures of uninfected MEFs maintained for the same time were used as controls. The data represent the mean of duplicates ± the standard error of the mean (SEM).

We next investigated whether infection in a different cell type, would alter JG and Col1.7G2 intracellular behavior. For this, we performed infections with JG and Col1.7G2 in immortalized mouse embryonic fibroblasts (MEFs) and evaluated parasite intracellular growth and oxidant production upon infection. Fig 7A shows the number of intracellular parasites per infected cell over a total period of 72 hours of infection in MEFs. As can be observed, there was no significant difference in the number of intracellular parasites between Col1.7G2 and JG in any of the analyzed points, being the growth curve of both *T*. *cruzi* populations similar to each other. In order to confirm that these cells did not respond to infection with oxidant generation, analysis of CM-H_2_DCFDA oxidization, 48 hours post infection with JG or Col1.7G2, was performed. For both cultures no statistically significant difference was observed in the levels of oxidized CM-H_2_DCFDA among control non-infected cultures and those infected with Col1.7G2 or JG (Fig 7B).

### JG epimastigotes present higher levels of intracellular Ca^2+^ and oxidant production upon treatment with H_2_O_2_

**Fig 8:**
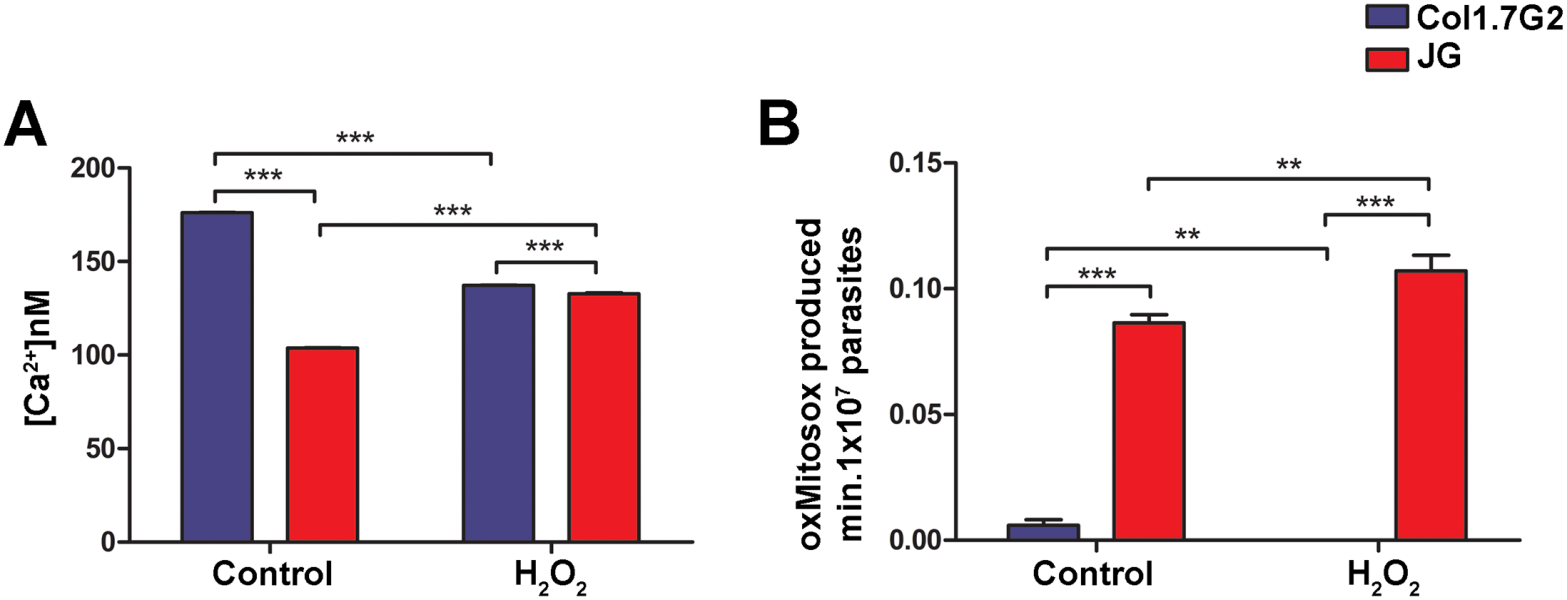
Quantification of epimastigotes intracellular levels of calcium (A) and superoxide anion (B) upon JG and Col1.7G2 epimastigotes treatment with H_2_O_2_. (A) JG and Col1.7G2 cells previously incubated with fura 2-AM, were treated or not (control) with 30 μM H_2_O_2_ for 30 minutes and the calcium levels in 10^7^ parasites/mL was determined. (B) JG and Col1.7G2 parasites were incubated with MitoSOX and subsequently exposed to 30 μM H_2_O_2_ for 30 minutes, the oxMitoSOX level was measured in 10^7^ parasites/mL. The data represent the mean of triplicates ± the standard error of the mean (SEM). Asterisks indicate statistically significant differences (**<0.01 *** p≤0.001—Student's t-test).

The above results suggest that oxidative stress may play in JG strain a role in the intracellular development of the parasite and may, in specific situations, be beneficial to its intracellular development. In the latter, oxidative stress could work as a signal triggering parasite intracellular growth [36, 37]. It has been shown in the literature that the increase in free intracellular Ca^2+^ levels in the cytoplasm of *T*. *cruzi* may represent an important signal, leading to an increase in the infective capacity of this parasite [33]. It has also recently been shown that the decrease of IP3 receptor expression in the parasite leads to a decrease not only in the infectivity, but also in parasite intracellular growth [38]. Thus, we decided to verify whether exposure of Col1.7G2 and JG to oxidative stress, by incubation with H_2_O_2_, could induce calcium signals in these parasites. Baseline levels of intracellular calcium, before treatment with H_2_O_2_ (control), were significantly higher for Col1.7G2, when compared to JG (Fig 8A). However, upon H_2_O_2_ treatment, only JG was capable of increasing the Ca^2+^ levels, as observed for *Trypanosoma brucei* [39] (Fig 8A).

Another important signaling molecule is superoxide radical (O_2_^•-^). It has been shown that high levels O_2_^•-^ is deleterious to cells, however in adequate concentrations O_2_^•-^ may function as a stimulator of cell growth, as well as to inhibit apoptotic pathways [40, 41]. Thus, we also investigated the influence of H_2_O_2_ treatment on O_2_^•-^/H_2_O_2_ levels in epimastigote forms of JG and Col1.7G2 following MitoSOX probe oxidation. Baseline levels of MitoSOX oxidation for Col1.7G2 were very low, significantly lower than those observed for JG (Fig 8B). On the other hand, oxidant treatment of JG led to an increase in MitoSOX oxidation (Fig 8B). The above result may indicate that oxidant treatment may, by some unknown mechanism, enhance parasite O_2_^•-^ production in the JG *T*. *cruzi* strain. Both intracellular O_2_^•-^ and or H_2_O_2_ may be in part, responsible for the cellular signaling that boost parasite proliferation.

## Discussion

One of the great questions regarding *T*. *cruzi* infection is what defines the development or not of serious clinical forms resulting from the infection. As mentioned previously, *T*. *cruzi* infection in humans has a very variable clinical course, in which infected individuals may be asymptomatic or even develop severe clinical symptoms, presenting cardiac, digestive or cardio-digestive disorders (reviewed by [42]). Understanding the mechanisms involved with the pathogenesis of this disease is essential for better control of the infection. Evidence from the literature shows that this clinical variability is related to a differential tissue tropism of parasite populations, which depends directly on the parasite-host cell interaction, without direct interference of the immune system [11, 13, 43, 44]. Cellular infection can be divided into two stages: invasion and intracellular multiplication. According to previous data from our group, intracellular multiplication seems to be of fundamental importance for the definition of this selection [14]. Studying the behavior of two clonal populations Col1.7G2 (*T*. *cruzi* I) and JG (*T*. *cruzi* II) of *T*. *cruzi* during infection in primary cultures of BALB/c embryonic cardiomyocytes it was observed that JG, a *T*. *cruzi* strain with strong tropism to BALB/c hearts, presented higher intracellular multiplication rates in these cells when compared to Col1.7G2 [11, 14].

In order to investigate the factors influencing this differential intracellular behavior we used as a study model the *in vitro* infection of primary cultures of BALB/c cardiomyocytes with the same *T*. *cruzi* clonal populations used in the previous studies, JG and Col1.7G2. However, this time the cardiomyocytes were isolated from neonatal mice. Regarding invasion rates, the data obtained here diverged from the data previously published by Andrade et al. (2010) [14]. This divergence may be related to the fact that, although from the same type of animal, the stage of differentiation of the cells was distinct from the work published earlier [14]. Cardiomyocytes isolated from neonatal mice may express distinct proteins from the ones obtained from mouse embryos, which could account for the differences in invasion rates observed for these two cells [45]. Nonetheless, in BALB/c neonatal cardiomyocytes, JG still presented a higher multiplication rate when compared to Col1.7G2. These data reinforce the idea that the intracellular development of the parasite, as suggested before [14], may be more important for the determination of *T*. *cruzi* tissue tropism than cellular invasion itself. This hypothesis is also supported by the data obtained here from JG and Col1.7G2 infections in human cardiomyocyte cultures derived from iPSCs. Upon infection in these cells, even though Col1.7G2 invasion rates were higher than JG, parasite intracellular growth was greater for JG infected cultures.

Several factors could affect the intracellular development of *T*. *cruzi*, among them its intracellular traffic. During cell infection, *T*. *cruzi* uses the cell membrane repair mechanism to promote its internalization in non-professional phagocytic cells, forming a vacuole containing lysosomal markers and content [46, 47]. The acidic content of the vacuole allows the parasite to gradually escape into the cytoplasm of the cell, where it completes its transformation into the amastigote form and initiates its intracellular multiplication [15, 48–51]. It was possible that a faster escape could advance the transformation of the parasite into the amastigote form and thus allow it to start its multiplication sooner. In fact, trans-sialidase superexpressor parasites, which escape faster from their parasitophorous vacuoles, differentiate into the amastigote earlier than wild type parasites [52]. Our data showed that, there was no difference in the rate of parasitophorous vacuole escape between Col1.7G2 and JG. Therefore this could not account for the differences in parasite intracellular development observed in the cardiomyocytes.

Data from the literature show that infected cells are able to respond to infection by activating several genes, which could interfere with the intracellular behavior of the parasite [19, 20, 22, 24]. Thus, we decided to evaluate the response of the host cell to infection, trying to correlate this data with the intracellular development of *T*. *cruzi*. For this, we investigated whether the production of ROS upon infection could be responsible for the differential intracellular growth of JG and Col1.7G2 in the studied cardiomyocyte cultures. It had already been shown that infection of cardiomyocytes with *T*. *cruzi* leads to a disturbance in the membrane potential of the mitochondria generating ROS [23]. In fact, by using the CM-H_2_DCFDA probe, we observed an increase in ROS production in primary cultures of BALB/c and human cardiomyocytes infected with Col1.7G2 (72 hours) or JG (48 and 72 hours) post infection, when compared to control uninfected cultures. Additionally, for JG infected cultures, a higher amount of ROS was produced 48 hours post infection, indicating a faster and stronger response of cardiomyocytes infected to this clonal population.

The high increase in ROS detected in BALB/c cardiomyocyte cultures 48 hours post infection with JG was likely generated by mitochondrial dysfunction as revealed by the analysis of the respiratory control index (RCI) in BALB/c cardiomyocyte infected cultures, although other sources cannot be ruled out. At this time post infection, cultures infected with JG presented a significant decrease in the RCI. The RCI is the best general measure of mitochondrial function in cell populations that have sufficiently active glycolysis to support metabolism, while mitochondrial function is manipulated [31]. Thus, a decrease in RCI does indicate mitochondrial dysfunction. These results are in agreement with previous data from the literature showing that upon infection with *T*. *cuzi* mitochondrial potential is disturbed, inducing ROS production [23]. On the other hand, we could not detect mitochondrial dysfunction in Col.17G2 infected cardiomyocytes 48 hours post infection. In agreement with this, at this time post infection, we could also not detect an increase in ROS in Col1.7G2 infected cardiomyocytes, when compared to control non-infected cultures. We are not sure why Col1.7G2 did not cause changes in cardiomyocyte mitochondrial RCI 48 hours post infection, but it may have to do with differences in the strains used. It is well known that *T*. *cruzi* populations do vary in their behavior during infection in cells, which may account for changes in their ability to interfere with mitochondrial function. In fact, although lower than that observed upon JG infection, we did observe changes in the RCI 72 hours post infection. So it is possible that upon infection with this strain there is a delay in the induction of mitochondrial dysfunction and generation of oxidative stress. In fact, there is data in the literature showing that hearts of BALB/c mice infected with Colombian strain do present a significant increase in the oxidative stress [53].

*T*. *cruzi* has a sophisticated system of antioxidant defenses to protect parasite from oxidative stress [54]. Interestingly, for epimastigote forms, none of the anti-oxidant enzymes evaluated were more expressed in JG when compared to Col1.7G2. In fact, when there was a difference in enzyme expression, the higher expression was found in Col1.7G2. This was also the case for parasites that were previously exposed to oxidative stress by incubation with H_2_O_2_, which had been already shown to induce an increase in anti-oxidant enzyme levels [55]. In fact, for Coll.72G parasites previously exposed to H_2_O_2_, we observed an increase in Fe-SODA and MPX levels, showing that H_2_O_2_ was effective in inducing an increase in enzyme expression. So far, JG induced more ROS production in cardiomyocytes and was likely to be more susceptible to this generated oxidative stress.

ROS has been shown to have a dual effect on cells. Although data from the literature show that an increase in ROS can compromise the intracellular growth of several pathogens, including *T*. *cruzi*, the opposite has also shown to be true [56–61]. It has recently been demonstrated that the oxidative stress generated by *T*. *cruzi* infection can lead to an increase in the replication rate of this parasite [36, 37]. One of the possible explanations for the increase in the rate of parasite replication upon induction of oxidative stress could be the bioavailability of iron for use by the parasite [36, 62]. It is known that iron is important for several metabolic events, such as DNA replication, mitochondrial respiration and anti-oxidant defense [63]. Thus, although amastigote forms of the parasite have been shown to be capable of binding and importing transferrin [64] in the intracellular environment, the concentration of this protein is very low. It is possible that free iron is more easily acquired in this way and then contributes to a better adaptation of this parasite to the intracellular environment. In the case of trypanosomatids, superoxide dismutases, important anti-oxidant enzymes, are iron-dependent [65]. Alternatively, the presence of oxidative stress could generate specific signals that would contribute to a more adequate response of the parasite in the cell, stimulating its faster replication. In fact, Finzi et al. (2004) also showed that pre-treatment of *T*. *cruzi* epimastigote forms with low concentrations of H_2_O_2_ increased parasite proliferation [66].

Considering the above, in our case, ROS seems to be important to give JG advantage during infection in cardiomyocytes. This is reinforced by the fact that inhibition of ROS by incubation of mouse and human cardiomyocytes with catalase inhibits JG intracellular growth. In fact, the inhibition of JG growth seemed even more prominent in human cardiomyocyte cultures. However distinct batches of catalase were used in the two experiments, which could account for this difference. Nonetheless, these results imply that at least for some strains ROS may be involved in parasite intracellular growth. Additionally, this is also corroborated by the data obtained from infected fibroblasts, which in our experimental conditions do not produce ROS in response to infection. In these cells JG did not present any growth advantage when compared to Col1.7G2. Previous studies have shown that a recombinant strain of *T*. *cruzi*, an *E*. *coli* MutT superexpressor (an enzyme involved in DNA repair), is more efficient in cell colonization compared to wild type parasites. In that work it was suggested that 8-oxo-GMP, generated by degradation of 8-oxo-GTP by MutT, could serve as signal to produce parasites more adapted to the intracellular environment [67]. It has also been shown that low concentrations of ROS were sufficient to promote better infection in *in vitro* and *in vivo* experiments [37]. Overall, our results suggest that ROS may have, in some specific circumstances, a helpful role in *T*. *cruzi* cell proliferation in non-professional phagocytes, which had not been shown before.

Recently, Vilar-Pereira and colleagues [53] have studied the role of antioxidants in cardiac function during *T*. *cruzi* infection in mice. For this they evaluated the production of ROS in the hearts of BALB/c mice infected with Colombian strain, by intravital microscopy at the chronic stage, before and after the treatment with different antioxidant agents. In this case, as mentioned before in this discussion, they found high amounts of oxidative stress in hearts of non-treated mice, possibly due to the fact that the experiments were performed *in vivo* at later time points, after several rounds of parasite infection. Additionally they showed electrical and mechanical dysfunction in these infected mice [53]. Treatment with different antioxidants was able to in fact improve hart function. However, they also demonstrated that only treatment with resveratrol was able to reduce parasite burden, but not treatment with the other antioxidant drugs. This is in contrast with previous findings from the same group showing that heart parasite burden is decreased in response to antioxidant treatment [36]. However in the latter they have used *T*. *cruzi* Y strain. Colombian and Y strain, such as the clone of Colombian strain (Col1.7G2) and JG used in our study, belong to two different *T*. *cruzi* lineages, *T*. *cruzi* I and II, respectively. It is possible that parasites from different lineages do respond differently to ROS. In this case, for *T*. *cruzi* II strains ROS may not have an effect in controlling or signaling to this parasite and that the effect of resveratrol may be by another signaling pathway, while *T*. *cruzi* I strains would be responsive to ROS. Our data supports this hypothesis since treatment does affect the *T*. *cruzi* I population, but not *T*. *cruzi* II. Whether *T*. *cruzi* II strains do not really respond to ROS or whether this response depends on the amount or the type of response triggered by the ROS production still remains to be elucidated.

There are several data in the literature showing that the presence of oxidative stress could generate signaling molecules. Here we show that parasite treatment with H_2_O_2_, leads to an increase in intracellular levels of calcium and also probably in O_2_^•-^ and/or H_2_O_2_ production in JG, but not in Col1.7G2 *T*. *cruzi* clone, reinforcing that JG is more responsive to ROS, at least in this condition. Both molecules have been shown to interfere with cell death and replication [41, 68]. In relation to calcium levels, it has been shown that in epimastigotes the regulation of intracellular calcium is important for multiplication and metacyclogenesis [69]. In the literature it has also been shown that ROS are capable of increasing intracellular Ca^2+^ in parasites such as *Trypanosoma brucei* [39]. To the best of our knowledge this is the first time that it is shown that *T*. *cruzi*, in this case JG strain, can also respond to oxidative stress by altering intracellular calcium levels. In this case, the increase in calcium levels observed for JG was not sufficient for induction of cell death, but could be important for signaling some pathway related to cell proliferation. With respect to O_2_^•-^, there are reports showing that its increase may induce programmed cell death in *T*. *cruzi* and that parasites overexpressing mitochondrial Fe-SODA are more resistant [57]. However, there are reports in the literature showing that an increase in the concentration of this molecule may also signal for increased cell proliferation, as well as to work as an inhibitor of apoptotic pathways [40, 41]. In *Dictyostelium discoideum*, for example, the overexpression of SOD, with consequent consumption of O_2_^•-^, leads to the inhibition of multicellular aggregates [41]. What would determine whether ROS is responsible for death or proliferation would certainly be related to the amount to which parasites are exposed and the ability of the parasite to sense and trigger the intracellular signaling.

The data presented in this work suggest a mechanism responsible for the better development of JG, dependent on the parasite response to oxidant production, in cardiomyocyte cultures and may contribute to the understanding of the behavior of *T*. *cruzi* populations during infection in the host.
